# Physicochemical, Pre-Clinical, and Biological Evaluation of Viscosity Optimized Sodium Iodide-Incorporated Paste

**DOI:** 10.3390/pharmaceutics15041072

**Published:** 2023-03-27

**Authors:** Soo-Jin Chang, Yu-Jin Kim, Huong Thu Vu, Ji-Myung Choi, Jeong-Hui Park, Seong-Jin Shin, Khandmaa Dashnyam, Jonathan C. Knowles, Hae-Hyoung Lee, Soo-Kyung Jun, Mi-Ran Han, Joon-Haeng Lee, Jong-Soo Kim, Ji-Sun Shin, Jong-Bin Kim, Jung-Hwan Lee

**Affiliations:** 1 Department of Pediatric Dentistry, College of Dentistry, Dankook University, 119 Dandaero, Cheonan 31116, Republic of Koreapedoshin@dankook.ac.kr (J.-S.S.); 2 Department of Biomaterials Science, College of Dentistry, Dankook University, 119 Dandaero, Cheonan 31116, Republic of Korea; 3 Institute of Tissue Regeneration Engineering (ITREN), Dankook University, 119 Dandaero, Cheonan 31116, Republic of Korea; 4 Department of Nanobiomedical Science & BK21 PLUS NBM Global Research Center for Regenerative Medicine, Dankook University, 119 Dandaero, Cheonan 31116, Republic of Korea; 5 Drug Research Institute, Mongolian Pharmaceutical University & Monos Group, Ulaanbaatar 14250, Mongolia; 6 UCL Eastman-Korea Dental Medicine Innovation Centre, Dankook University, 119 Dandaero, Cheonan 31116, Republic of Korea; 7 Cell & Matter Institute, Dankook University, Cheonan 31116, Republic of Korea; 8 Division of Biomaterials and Tissue Engineering, Eastman Dental Institute, Royal Free Hospital, Rowland Hill Street, London NW3 2PF, UK; 9 Department of Dental Hygiene, Hanseo University, 46 Hanseo 1ro, Seosan 31962, Republic of Korea; 10 Mechanobiology Dental Medicine Research Center, Cheonan 31116, Republic of Korea

**Keywords:** therapeutic dental paste, sodium iodide, iodoform, root filling dental material, viscosity

## Abstract

This study aimed to investigate the impact of different viscosities of silicone oil on the physicochemical, pre-clinical usability, and biological properties of a sodium iodide paste. Six different paste groups were created by mixing therapeutic molecules, sodium iodide (D30) and iodoform (I30), with calcium hydroxide and one of the three different viscosities of silicone oil (high (H), medium (M), and low (L)). The study evaluated the performance of these groups, including I30H, I30M, I30L, D30H, D30M, and D30L, using multiple parameters such as flow, film thickness, pH, viscosity, and injectability, with statistical analysis (*p* < 0.05). Remarkably, the D30L group demonstrated superior outcomes compared to the conventional iodoform counterpart, including a significant reduction in osteoclast formation, as examined through TRAP, c-FOS, NFATc1, and Cathepsin K (*p* < 0.05). Additionally, mRNA sequencing showed that the I30L group exhibited increased expression of inflammatory genes with upregulated cytokines compared to the D30L group. These findings suggest that the optimized viscosity of the sodium iodide paste (D30L) may lead to clinically favorable outcomes, such as slower root resorption, when used in primary teeth. Overall, the results of this study suggest that the D30L group shows the most satisfactory outcomes, which may be a promising root-filling material that could replace conventional iodoform-based pastes.

## 1. Introduction

Over the years, several types of root canal filling materials have been developed and studied to improve the effectiveness of treatments for primary teeth. Among these, zinc oxide and eugenol (ZOE) was widely used for pulpectomies in primary teeth in the past; however, it is no longer commonly used due to their limited antibacterial properties, slow absorption, and tendency to cause irritation to apical tissues [1]. Moreover, even after physiologic root resorption, ZOE may leave particles in the periapical tissue.

To overcome these issues, iodoform-based or calcium hydroxide-containing materials have been introduced, which are known for their ability to easily resorb if pushed beyond the apex, exhibit no foreign body reaction, and possess potent germicidal properties [2,3,4,5]. Several such materials have been developed, and among them, Vitapex^®^, a premixed paste consisting of calcium hydroxide, silicone oil, and iodoform, is considered to be an excellent option for filling root canals in primary teeth, among other materials available. Although iodoform-based pastes have been found to possess advantageous features [6], it has been observed that this paste tends to resorb at a faster rate than the physiologic rate of root resorption, suggesting that it may hasten the exfoliation of primary tooth [7,8]. Such early exfoliation of primary teeth can lead to complications, such as the impaction of the permanent successor or shifting of the adjacent tooth. A study by Moskovitz et al. suggested that the accelerated root resorption of primary molars following root canal treatment may result from irritation of the tissue surrounding the root apex by the root canal filling material. The formation of odontoclasts, which cause external root resorption, can be instigated by products derived from infection and necrosis of the periradicular tissue. The study posits that irritation of the dental follicle by the iodoform-containing root canal filling paste may be the cause of the accelerated root resorption [9]. The initiation and pace of root resorption are influenced by various local factors. Among these, inflammation caused by decay, pulp necrosis, and pulp treatment can hasten the process of root resorption [10,11]. The relationship between accelerated root resorption and iodoform-containing root canal filling material remains unclear, and the accelerated resorption may be a consequence of the inflammatory process initiated by irreversible pulpitis. Further studies are necessary to explore the relationship between root canal filling material and the rate of root resorption. In light of concerns surrounding the accelerated root resorption associated with iodoform-based pastes, a previous study aimed to address these limitations by examining the use of sodium iodide as a potential alternative. By replacing iodoform with sodium iodide, the study sought to evaluate the impact of this modified paste on the rate of root resorption (NaI) [12]. The sodium iodide-based paste was formulated by combining equal amounts of calcium hydroxide, silicone oil, and NaI. An iodoform-based paste was also prepared for the control group, with iodoform used instead of NaI. The investigation revealed that the sodium iodide-based paste was associated with a lower degree of osteoclast differentiation in comparison to the iodoform-based paste, indicating that NaI may be a more effective means of reducing the rate of accelerated root resorption than iodoform [12]. Although NaI may be considered an appropriate filling paste for primary teeth, several limitations exist due to difficulty in material insertion since it is not injectable. The paste should be syringeable with thin needle tips to facilitate clinical use [13].

The ideal material should possess certain characteristics such as flowability to fill the intricate root canal system, biocompatibility, radiopacity, and disinfectant properties [5]. Evaluating these properties is essential to ensure the clinical effectiveness and handling characteristics of the paste. Polydimethylsiloxane (PDMS) is a synthetic polymer classified under the polysiloxane group of silicones, commonly known as silicones. It is transparent, chemically inert, hydrophobic, and non-flammable. Its rheological properties, particularly its viscoelasticity, make it stand out as an exceptional flow agent, especially at elevated temperatures [14,15]. In dentistry, PDMS has found applications as a hydrophobic coating for tooth enamel and metallic dental implants, enhancing their antibacterial properties [16]. Additionally, it serves as a separating agent, lubricant, and component of impression materials. Silicone oil is frequently used to improve the flow properties and reduce the surface tension of impression materials [17]. Furthermore, it has been employed as a filling material additive to improve handling properties and decrease viscosity [18]. In this study, we aimed to develop an appropriate syringeable paste for primary teeth by examining the physicochemical and biological properties of three different viscosities of silicone oil mixed with calcium hydroxide and either iodoform or sodium iodide. The null hypotheses were:

1. Pastes with three different silicone oil viscosities will not show a difference in physicochemical properties.
2. Compared to iodoform-based paste, sodium iodide-based paste will demonstrate similar osteoclast formation.

## 2. Materials and Methods

### 2.1. Sample Preparation

For this study, an iodoform (Alfa Aesar, Heysham, UK), calcium hydroxide (Sigma–Aldrich, Burlington, MA, USA), and silicone oil were mixed on a glass plate with a sterilized spatula at a proportion of 1:1:1, respectively, to fabricate an iodoform-based paste. Sodium iodide-based paste was created by mixing equal proportions of NaI (Sigma–Aldrich, Burlington, MA, USA), calcium hydroxide, and silicone oil. The iodoform-based paste was denoted as I30; the NaI-based paste was designated as D30 for this study. Groups were divided based on D30 and I30 and subdivided into three groups based on the viscosities of silicone oil as high (H), medium (M), and low (L) to compare six groups: I30H, I30M, I30L, D30H, D30M, and D30L. Calcium hydroxide, silicone oil H (Sylgard 184, Dow Corning Co., Midland, MI, USA), silicone oil M (Shin-Etsu Silicone KF-96 1000 cst, Shin-Etsu Chemical Co., Tokyo, Japan), and silicone oil L (Shin-Etsu Silicone KF-96 350 cst, Shin-Etsu Chemical Co., Tokyo, Japan) were used. All materials were mixed by the same operator, as shown in Figure S1.

### 2.2. Physicochemical Characteristics of Samples

Flow, film thickness, radiopacity, and solubility tests for the control and experimental groups followed International Standard Organization (ISO) 6876:2012 (Dentistry—Root Canal Sealing Materials) protocol. All experiments were performed at room temperature (23 ± 2 °C) and 5% relative humidity unless otherwise noted. All experiments were performed in triplicate, and the mean values were used for comparisons.

#### 2.2.1. Flow

The experiment involved applying 0.05 mL of mixed paste onto a glass plate with a diameter of 40 mm and height of 5 mm. Three minutes after the start of mixing, another glass plate (20 g) of the same size and height was placed with an additional 100 g mass for 7 min. After 10 min, the maximum and minimum diameter was calibrated using a digital caliper (Mitutoyo, Mitutoyo Corp., Kawasaki, Japan). This experiment was performed equally thrice with three samples to calculate the average and standard deviation.

#### 2.2.2. Film Thickness

Two glass plates (15 mm width, 15 mm length, and 5 mm height) were placed together, and their thickness was measured using a micrometer (Mitutoyo, Mitutoyo Corp., Kawasaki, Japan). 0.01 mL mixed specimens were placed on one of these plates; the other plate was placed on top of this plate. After 3 min, a weight of 150 N was applied, ensuring that all specimens filled the glass plate. After 10 min, the thickness of the two glass plates with specimens was measured using a micrometer. Film thickness was calculated by subtracting the thickness of two glass plates with specimens from those without specimens. This experiment was performed equally thrice to calculate the average and standard deviation.

#### 2.2.3. Radiopacity

A Teflon mold (diameter 10 mm and thickness 1 mm) was prepared for placing the specimen. Specimens and an aluminum step wedge were placed on an X-ray film (Kodak Insight, Rochester, NY, USA) for digital radiography. Kodak-2200 X-ray was used (Kodak Insight, Rochester, NY, USA) under 70 kV, 7 mA, and 0.3 s with a 300 mm distance from film to X-ray. Images were analyzed using the Grayscale program (ImageJ version 1.53a, National Institutes of Health, Bethesda, MD, USA). The optical density values for the specimens were compared using different radiopacities of aluminum wedge thickness. The optical density values for the specimens were converted to aluminum wedge thickness using a formula from Hungaro Duarte et al. [19].

#### 2.2.4. Optical Images

To compare the surface characteristics of the materials, a glass plate was coated with 0.01 mL of paste and examined under a 25× microscope (S39A, Microscopes Instrument, Suwon, Korea).

#### 2.2.5. Solubility

The solubility was measured by taking 1 g of mixed pastes and placing it in a Teflon (polytetrafluoroethylene; DuPont, HABIA, Knivsta, Sweden) ring mold with an internal diameter of 20 mm and a height of 1.5 mm, and then removing any excess. The initial mass of specimen (I0) was measured to the nearest 0.001 g. Two filled molds were immersed in a petri dish with 50 mL distilled water and then placed in the cabinet under 100% relative humidity at 37 ± 1 °C for 24 h. After 24 h, 50 mL solution from the specimen was filtered into a funnel and placed in a dry oven at 80 ± 2 °C for complete drying. The weight of the empty beaker was measured (I1) before the solution was filtered. Subsequently, the desiccator was cooled down and weighed in order to establish the final mass (I2) of each specimen. The difference in mass was then computed using the following equation.

(1) $$\mathrm{Solubility}=\frac{\mathrm{Final}\;\mathrm{weight}\;\mathrm{of}\;\mathrm{beaker}\;(I2)\;-\;\mathrm{initial}\;\mathrm{weight}\;\mathrm{of}\;\mathrm{beaker}\;(I1)}{\mathrm{initial}\;\mathrm{weight}\;\mathrm{of}\;\mathrm{sample}\;(I0)}\;\times \;100\;(\%)$$

#### 2.2.6. Extraction Analysis

For analysis of ion release and pH variation, approximately 1 ± 0.1 g of paste was filled with 5500 µL distilled water and placed in a shaking incubator under 37 ± 1 °C for 24 h. After 24 h, the solution was filtered through a 0.22-µm filter (Corning, Corning, NY, USA) and subjected to inductively coupled plasma-atomic emission spectrometry (Optima 8300, PerkinElmer, Waltham, MA, USA), ion chromatography, and pH tests. The pH meter employed in this study was the Orion VERSA Star Pro (Thermo Fisher Scientific, Waltham, MA, USA), which was calibrated with buffer solutions of pH 4.01, 7.00, and 10.01. The same solutions used to test the pH variation were used to test for ion release [20]. Measurements for pH and ion release were repeated thrice; mean and standard deviations were used.

#### 2.2.7. Viscosity

A Discovery HR-1 instrument (TRIOS, TA Instruments, New Castle, DE, USA) was used for the viscosity test. 0.3 mL of pastes were spread evenly on the Peltier plate (diameter 60.0 mm). The upper aluminum plate was moved downward to adjust the gap to 500 µm, and the excess paste was removed. All analyses were performed at room temperature with a 0.05 to 1000 Hz frequency [21]. A graph was plotted with the X-axis as the frequency (Hz) and the Y-axis as the complex viscosity (Pa.s).

#### 2.2.8. Injectability

The mechanical testing setup was prepared using a manufactured mold that fits a 1 mL syringe for the injectability test. The paste was pushed at 0.05 mm/s until it extruded from the needle tip [22].

#### 2.2.9. Endodontic Treatment

##### Conventional Root Canal Treatment

To analyze filling capacity, 12 artificial mandibular premolars were endodontically treated. The teeth were divided into four groups based on pastes (I30M, I30L, D30M, D30L); I30H and D30H were excluded because they could not be extruded from the syringe needle tip by the operator. To determine the length of the root canal, a diagnostic radiograph was taken with a K file inserted into each canal. The root canal working length was estimated from the preoperative periapical radiograph and determined to be 1 mm short of the radiographic apex. Root canals were gently debrided with K files up to #35 and thoroughly irrigated with normal saline [23,24]. After drying with paper points, the paste was transported to the canals directly from the syringe. The syringe was inserted into the canal near the apex and slowly withdrawn when the paste flowed back into the pulp chamber [25]. Postoperative periapical radiographs were taken.

##### Removability of Pastes

Pastes were removed using an ultrasonic scaler; the tip was inserted till it did not go further into the root canal. The scaler was used for 1 min, and the removability of the paste was evaluated using periapical radiographs.

### 2.3. Biological Characteristics of Samples

In the context of the present investigation, samples I30L and D30L were designated as representative specimens and were consequently chosen for employment in subsequent experimental analyses. Two grams of paste with 10 *mL* α-MEM (Minimum Essential Medium Eagle, Alpha Modification, with L-glutamine, ribonucleosides and deoxyribonucleosides, sodium bicarbonate, and without L-ascorbic acid; Welgene, Gyeongsan, Korea) was placed in an incubator at 37 °C and 5% CO_2_ for 24 h. The solution was filtered through a 0.22 µm pore syringe filter (Corning, Corning, NY, USA). The extracted media was diluted into different ratios: 0%, 6.25%, 12.5%, 25%, 50%, and 100% in α-MEM. A 1% penicillin/streptomycin (PS; Gibco, Thermo, Fisher Scientific, Hampton, NH, USA) and 10% fetal bovine serum (FBS; Corning) were added into the diluted media; α-MEM supplemented with 1% PS and 10% FBS was prepared as control media. The final media was used for further cytotoxicity tests and osteoclast differentiation assay.

#### 2.3.1. Cytotoxicity Test

A 96-well plate (uncoated, SPL Life Sciences, Gyeonggi, Korea) with RAW 264.7 cells at a density of 2 × 10^4^ cells/well (α-MEM, 10% FBS, and 1% PS) was incubated overnight to enable cell adhesion. Then, the cultured media was shifted to the extracts at different concentrations for the next 24 h. After 24 h of incubation, the medium containing 10% CCK-8 (cell counting kit 8, Dojindo, Japan) solution was replaced in each well, followed by incubation at 37 °C, 5% CO_2,_ and 95% humidity [26]. After 2 h of incubation under light protection, slightly yellow-colored (WST-8) cultured media turned to orange (formazan WST-8) through cellular activity. The level of dehydrogenase activity in cells determines the intensity of the orange color, which can be utilized to ascertain cell viability and the quantity of living cells. To measure the absorbance at 450 nm, a microplate reader (Varioskan^TM^ LUX, Thermo Fisher Scientific) was used. The absorbance reading obtained from each well was entered in the equation below to calculate the cell survival rate:

(2) $$Cell\;viability\left(\%\right)=\frac{OD\left(experiment\right)-OD\left(blank\right)}{OD\left(control\right)-OD\left(blank\right)}\times 100$$

The effect of the material extract on cell survival was also examined by staining live and dead cells (Live and Dead*^®^* Viability Kit, 0.5 µM calcein AM and 2 µM ethidium homodimer-1 solutions, Varioskan^TM^ LUX, Thermo Fisher Scientific, Waltham, MA, USA), and images were taken using a fluorescence microscope (IX71, Olympus, Tokyo, Japan). Live cells were determined by green fluorescent calcein-AM converted by intracellular esterase activity, and dead cells were discriminated by red-fluorescent ethidium homodimer-1 binding to exposed DNA. All experiments were replicated with six samples (*n* = 6).

#### 2.3.2. Osteoclast Differentiation Assay

The diluted extracts with concentrations of 12.5% and 25% were chosen for further experiments due to their observed safety in terms of cytotoxicity. To evaluate the effect of materials on stimulating osteoclast activity in bone resorption, RAW 264.7 cells, a well-known macrophage cell line with the capacity of active osteoclast differentiation in the presence of receptor activator of nuclear factors κB ligand (RANKL*),* were used [27]. Briefly, cells were seeded at a density of 2 × 10^4^ cells/well in an uncoated 24-well plate (SPL Life Sciences) in DMEM (Dulbecco’s Modified Eagle’s Medium, high glucose, with L D-glucose, L-glutamine, and sodium pyruvate, Welgen) supplemented with 10% FBS and 1% PS at 37 °C for 24 h. Next, the media was replaced with diluted extracts of either 12.5 or 25% concentration containing osteoclast differentiation supplements, including 50 ng/mL RANKL (Peprotech, Middlesex County, NJ, USA), 10 ng/mL M-CSF (Peprotech), and 1 ng/mL TGF-β2 (R&D Systems, Minneapolis, MN, USA). This procedure was repeated every 2 days for 6 days.

The differentiation effect was monitored by the level of osteoclast gene expression on days 2, 3, and 4 by quantitative real-time polymerase chain reaction (qRT-PCR). After cell extraction, total RNA was extracted using Ribospin^TM^ (cat. no. 304-150, GeneAll Biotechnology, Seoul, Republic of Korea). Following cDNA reverse transcription, the expression levels of cathepsin K, nuclear factor-activated T cells c1 (NFATc1), TRAP, and c-Fos genes that are closely associated with osteoclast differentiation were identified through the employment of the SensiMix SYBR Hi-ROX Mastermix (QT-605-05, Bioline) protocol on the StepOnePlus real-time PCR system (Applied Biosystems, Foster City, CA, USA). Glyceraldehyde-3-phosphate dehydrogenase (GAPDH) was used as the housekeeping gene. The qRT-PCR primers and their sequences are described in our previous study [12]. The RAW 264.7 cells were cultured in differentiation media without extracts, which served as the control. The results are expressed as relative expression of mRNA. All experiments were replicated with three samples (*n* = 3).

Following 6 days of culturing, the number of differentiated osteoclasts was determined through tartrate-resistant acid phosphatase staining (TRAP Staining Kit, Cosmo Bio, Tokyo, Japan), while actin ring formation was determined by phalloidin (Alexa Fluor™ 594, Thermo Fisher) and DAPI (4′,6-diamidino-2-phenylindole, Thermo Fisher) staining. Briefly, cells were fixed with 10% formalin neutral buffer solution (Tech & Innovation, Gangwon, Korea) for 30 min at room temperature and stained with TRAP solution at 37 °C. After 30 min, cells were washed with distilled water and stained with phalloidin and DAPI. Only the cells with more than 3 nuclei were counted. Stained cells were analyzed and captured using a light microscope (IX71, Olympus, Tokyo, Japan).

#### 2.3.3. mRNA Sequencing

The total RNA of differentiated RAW cells cultured in control media extracted I30L. D30L for 3 days was isolated using Trizol reagent (Invitrogen, Waltham, MA, USA). Significant genes in the I30L group were compared to the control and D30L groups and selected with three conditions: fold-changes *≥* 1.2, normalized data (log2) > 4, and *p* < 0.05 using ExDEGA software (v4.0.3, E-biogen Inc., Seoul, Korea). with raw data provided by E-biogen. The enriched functional annotation terms were analyzed with the above data using DAVID and visualized with ExDEGA GraphicPlus (E-biogen). A heatmap of hierarchical clustering was generated using MeV version 4.9.0.

- λ Library preparation and sequencing

The isolated mRNAs were utilized for cDNA synthesis in accordance with the manufacturer’s guidelines, followed by indexing through Illumina indexes 1–12. The enrichment step was performed using PCR. Subsequently, libraries were checked using the TapeStation HS D1000 Screen Tape (Agilent Technologies, Amstelveen, The Netherlands) to evaluate the mean fragment size. The quantification process was conducted with the aid of a StepOne Real-Time PCR System (Life Technologies, Inc., Carlsbad, CA, USA) using a library quantification kit.

- λ Data analysis

To ensure the quality of the raw sequencing data, FastQC (Hannon Lab., Jinan, China) was utilized. The removal of low quality reads (<Q20) and adapters were conducted with the aid of FASTX_Trimmer [28] and BBMap [29]. TopHat was employed to map the trimmed reads to the reference genome [30]. FPKM + Geometric normalization method was utilized for processing RC (Read Count) data, and EdgeR within R was utilized for this purpose [31]. FPKM (Fragments Per kb per Million reads) values were estimated using Cufflinks [32]. Data mining and graphic visualization were performed using ExDEGA (E-biogen Inc., Korea).

### 2.4. Statistical Analysis

Data are presented as the means ± standard deviations (SDs) and analyzed using SPSS software 26.0 (SPSS Inc., Chicago, IL, USA). One-way analysis of variance (ANOVA) was used with Tuckey’s multiple comparison test to evaluate data distribution within groups. All *p* < 0.05 was considered significant, and ‘ns’ indicates no significance.

## 3. Results

### 3.1. Physicochemical Properties

**Figure 1:**
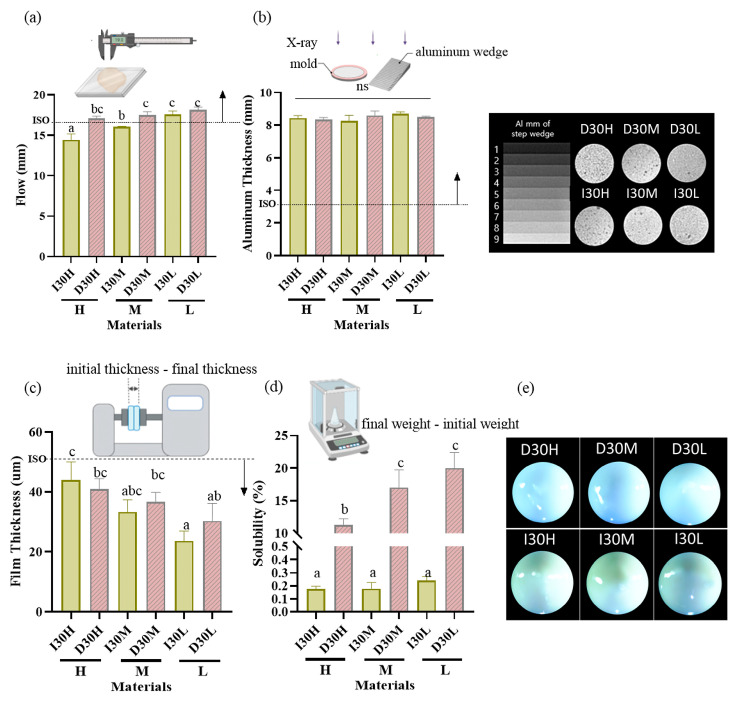
Physical properties of iodoform-based and NaI-based pastes (I30 and D30). (**a**) Flowability. All values except I30H and I30M were above 17 mm (ISO standard). (**b**) Radiopacity. All the aluminum thickness values were above the 3 mm Al (ISO standard). (**c**) Film Thickness. All groups met the ISO standard, which is under 50 µm. (**d**) Solubility. The solubility of D30 was higher than I30. (**e**) Optical microscopy images depicting the D30 and I30 groups, distinguished by their colors. The D30 group appears blue-green, whereas the I30 group appears yellow to white. Arrows indicate ISO 68762012 standards. Different superscript letters mean statistically significant differences among groups while ns mean not significant. Error bars mean standard deviations (*p* < 0.05).

**Figure 2:**
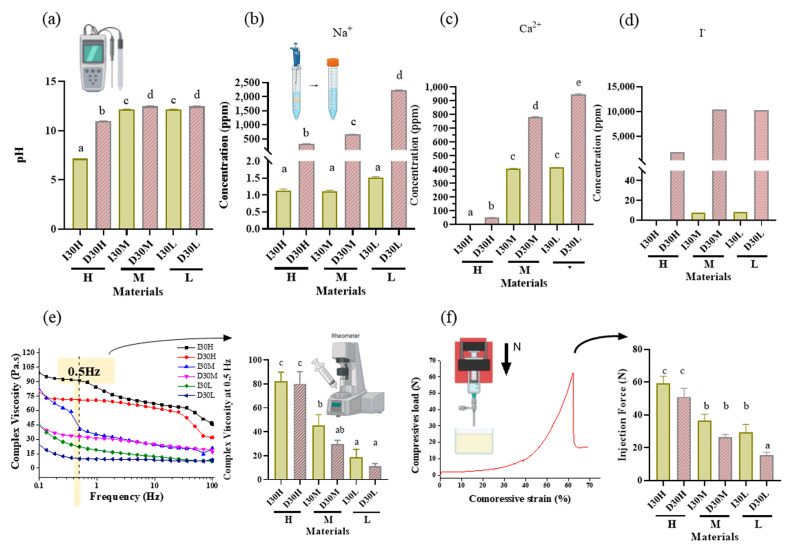
pH analysis and extraction analysis of iodoform-based and NaI-based paste (I30 and D30). (**a**) The pH was higher in the D30 groups than in the I30 groups. (**b**,**c**) Sodium and calcium ions were extracted using inductively coupled plasma atomic spectrometry (ICP/AES). The sodium and calcium concentrations were higher in the D30 group than in the I30 group. (**d**) Iodoform extracted by ion chromatography. The concentrations of iodoform were higher in the D30 group than in the I30 group. (**e**) Overall viscosity in the linear graph and viscosity at 0.5 Hz is shown in the bar graph. The viscosity of the I30 group was higher than that of the D30 group. Silicone oil with the L group showed the lowest viscosity compared to H and M silicone oil groups. (**f**) The highest compressive load is the site when the material extrudes out of the syringe. This peak load is shown in the bar graph. The injection force of the D30 group was lower than the I30 group. Silicone oil with the L group showed the lowest injection force compared to H and M silicone oil groups. Different superscript letters mean statistically significant differences among groups. Error bars mean standard deviations (*p* < 0.05).

The results of the physical properties are summarized in Table 1, Figure 1 and Figure 2. All pastes (except I30H and I30M) met or exceeded the minimum value of 17 mm required by ISO standards for the flowability test. Compared to the I30 groups, the D30 groups showed a higher flow rate. Statistical analysis revealed that D30L had flow values significantly superior to the other pastes tested (*p* < 0.05) (Figure 1a). All groups met the requirement of the ISO 6876 protocol for radiopacity (Figure 1b). There was no significant difference among H, M, and L silicone oil groups. The radiopacity values for iodoform and sodium iodide were similar. All pastes met ISO standards for the film thickness test, with the maximum value being 50 µm (Figure 1c). Statistical analysis revealed that silicone oil with H showed film thickness significantly superior to the other pastes tested (*p* < 0.05). For the solubility test, compared to the I30 group, the D30 group showed higher solubility; sodium iodide paste showed significantly higher solubility than iodoform paste (*p* < 0.05) (Figure 1d). We used optical imaging to visualize evenly blended calcium hydroxide and silicone oil with NaI or iodoform. Iodoform pastes showed yellowish surface color, while sodium iodide pastes showed bluish-grey color (Figure 1e).

**Figure 3:**
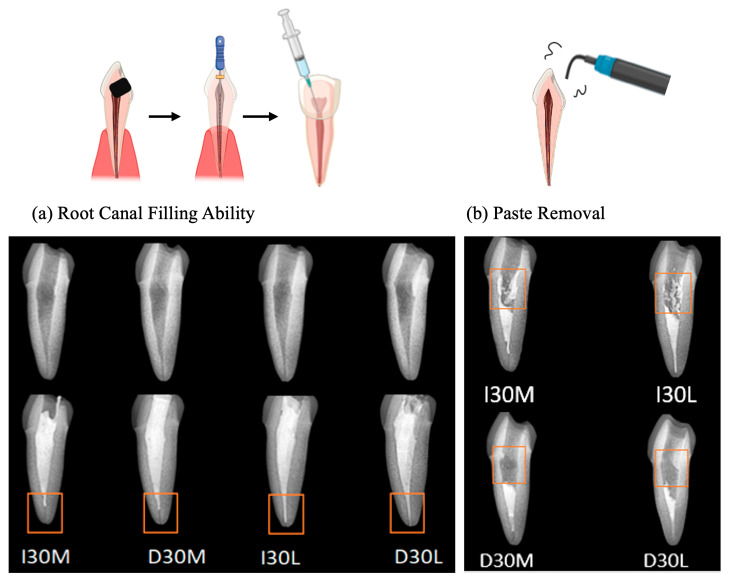
Usability test of NaI-based paste compared to iodoform-based paste. (**a**) Postoperative radiograph immediately after root canal treatment. (**b**) Postoperative radiograph after removing filling material using an ultrasonic scaler.

As the viscosity of silicone oil decreased, pastes showed alkaline pH, higher solubility, and higher release of sodium and calcium ions (Figure 2a). The amount of sodium and calcium ion release was significantly different, with the highest values observed for D30L (Figure 2b,c). Furthermore, silicone oil with the L group showed the highest release of iodoform (Figure 2d). For the viscosity test, an increase in frequency was associated with decreased complex viscosity for all materials (Figure 2e). Complex viscosity difference at the frequencies of 0.5 Hz was the highest in I30H, and the lowest was D30L. The L group showed the lowest viscosity compared to H and M groups. Material viscosity decreased with reduced injection velocity (Figure 2f). I30 and D30 showed no significant difference; however, silicone oil L showed the lowest injection force out of the syringe needle tip. Different lowercase superscript letters indicate a significant difference between groups. For root canal filling treatment, an artificial tooth was used; evaluation of the quality of root filling was based on the immediate postoperative radiograph. Compared to the M group, L group material penetrated deeper into the root canal (Figure 3a,b).

### 3.2. Effect of Material Extracts on Cell Viability

**Figure 4:**
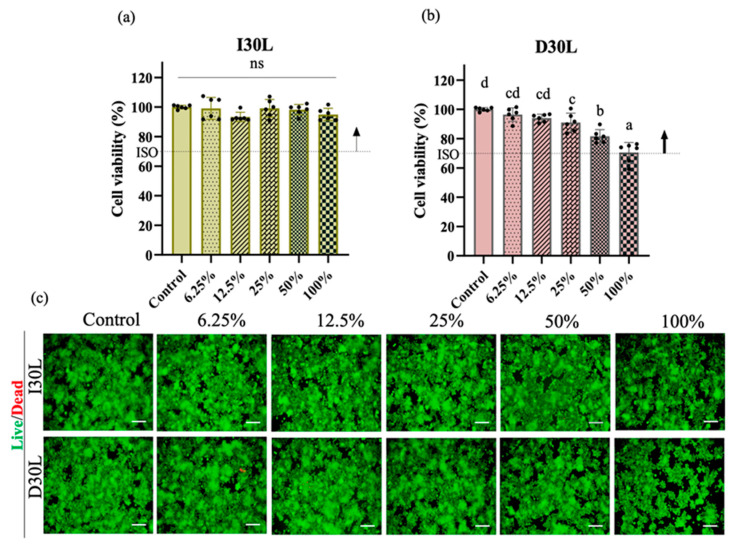
Osteoclast precursors viability test of NaI-based paste using extract compared to iodoform-based paste. (**a**,**b**) Cytotoxicity of the extract of NaI-based paste and iodoform-based paste using osteoclast precursors and extracted solutions of different concentrations (control, 12.5, 25, 50, 100%) for 24 h. Arrows indicate ISO standards with the dashed line (70%) represents the cutoff level established by the Cytotoxicity ISO (10993-5), (*n* = 6, *p* < 0.05). Both I30L and D30L met ISO recommendations up to 50% concentration in cell viability, but at 100% concentration, the cell viability decreased slightly at D30L. Different superscript letters mean statistically significant differences among groups while ns mean not significant. Error bars mean standard deviations (*p* < 0.05). (**c**) Representative images of live/dead staining show live cells stained green and dead cells stained red. The 100% extract from the D30 group displayed a lower count of live cells compared to other groups, as observed in the images.

The effect of material extracts on RAW 264.7 cell viability was assessed after 24 h of treatment. Exposure of RAW 264.7 cells in different concentrations of I30L extract did not induce cell death compared to cells cultured in growth media (*p* > 0.05). The cell toxicity of D30L extract is dependent on its concentration. At low concentrations (<25%), the D30L extract does not seem to induce toxicity (*p* > 0.05), while at higher concentrations (50%), it could be toxic to RAW 264.7 cells (*p* < 0.05). However, the D30L extract satisfied the Cytotoxicity ISO standard requirement (10993-5) for in vitro cytotoxicity of medical materials, with about 70% cell survival in the presence of 100% D30L. The live/dead assay results were consistent with the CCK-8 assay. Figure 4a, and b shows the cell viability results of the material extracts at various concentrations (control, 12.5%, 25%, 50%, and 100%). There was a decrease in cell viability at 100% extract of D30L compared to the 0% extract (*p* ˂ 0.05). The I30L extracts did not induce cell toxicity compared to the D30L groups, with no significant difference in cell viability at different percentages of extracts. To verify cell survival, cell images were captured following staining for live/dead cells. The fluorescence microscopy images of live/dead cells were utilized to re-evaluate the cytotoxicity of I30L and D30L against RAW 246.7 cells using CCK-8 (Figure 4c). Green and red stains were utilized to differentiate live cells from dead cells. Micrographs of stained cells revealed a lower count of viable cells in the 50% extract of the D30 group, compared to the I30 group, thus substantiating the outcomes of the cell viability assay. Based on the results obtained from the CCK-8 and live/dead cell staining, it was observed that an extract concentration of 25% extract for both I30 and D30 materials did not induce toxicity in the RAW 264.7 cell culture. Therefore, we selected this concentration of extracts for subsequent experiments. For verifying the effects of the material-extracted solutions on the expression of genes related to osteoclast differentiation from RAW 264.7 cells, the mRNA expression levels were evaluated by qPCR at three time points (days 2, 3, and 4).

### 3.3. Effect of Material Extract on Osteoclast Differentiation

We performed an osteoclast differentiation assay using 12.5% and 25% I30L and D30L, respectively, for 6 days of induction. The osteoclast differentiation effect of the extract was evaluated by the gene expression of osteoclast markers (qRT-PCR), TRAP, phalloidin, and DAPI staining. Cells were collected to perform qPCR on days 2, 3, and 4 of the differentiation process. 

**Figure 5:**
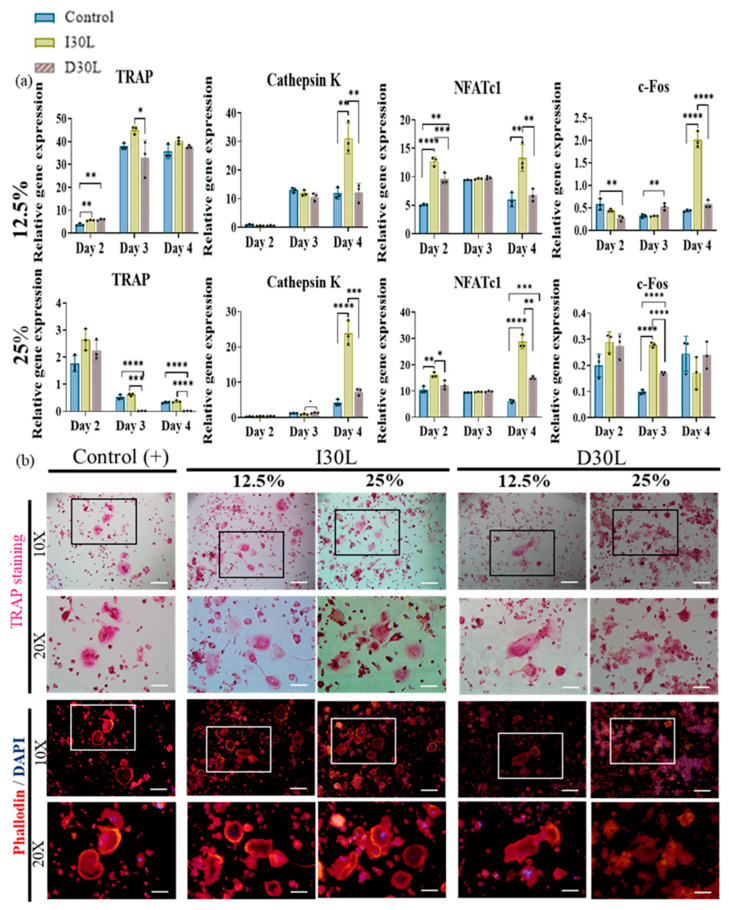
Osteoclast differentiation test of NaI-based paste using extract compared to iodoform-based paste. (**a**) RAW 264.7 cells were differentiated into osteoclasts in 12.5% and 25% nontoxic extracts from culture media. After 2, 3, and 4 days of culture, the gene expression of c-FOS, NFATc1, cathepsin K, and TRAP were measured by qPCR. * represents *p* < 0.05, ** *p* < 0.01, *** *p* < 0.001, **** *p* < 0.0001, *n* = 3. (**b**) Representative images of TRAP and phalloidin & DAPI staining of osteoclasts from one of the three experiments are shown. The scale bar is written and the 10×-scale bar is 200 μm and the 20×-scale bar is 100 μm. TRAP staining showed the highest number of differentiated osteoclasts in I30L and the lowest in D30L. The size of the actin ring increased in I30L.

In the present study, we observed that TRAP upregulation at a concentration of 12.5% was evident on day 2, followed by a significant increase on day 3, and a slight decline on day 4 across all groups. Additionally, the I30 group exhibited a substantial increase in cathepsin K expression on day 4 compared to the control and D30L groups. Furthermore, NFATc1, a key modulator of osteoclast maturation, was upregulated in most groups, with the I30 group showing significantly higher expression than the D30 and control groups. Finally, the c-Fos, a transcription factor known to regulate NFATc1, increased significantly in I30 on day 4 compared to D30 and the control group (Figure 5a).

In 25% concentration, TRAP declined significantly in D30L compared to I30L and the control group. Cathepsin K increased significantly in the I30L group on day 4 compared to D30L and control groups. NFATc1 showed high expression in I30L compared to D30L and the control group. For c-Fos, D30L and the control group decreased on day 3, while it increased on day 4. I30L increased on day 3 and decreased on day 4 (Figure 5a). 

TRAP and actin ring staining assays, which are commonly used as biomarkers of osteoclasts, were utilized to measure the impact of the materials on osteoclast formation. RAW 264.7 cells were cultured in osteoclast differentiation media for 6 days and then stained for tartrate-resistant acid phosphatase in osteoclasts (TRAP stain) and its actin rings (phalloidin and DAPI). Cells were fixed with 4% paraformaldehyde followed by TRAP and phalloidin & DAPI staining (actin ring staining). The number of TRAP-stained osteoclasts was highest in 12.5% and 25% I30L extracts, followed by the control group. Cells cultured in 12.5% and 25% D30L could inhibit osteoclast formation when the number of osteoclasts formed was lower than both I30L and the control group. As seen in the actin ring formation, as shown in Figure 5b, stimulation with the extracts of I30L did not increase the size of the actin rings compared to the control group, while the size of the actin rings in the 25% D30L was smaller than remaining groups. 

### 3.4. mRNA Sequencing

**Figure 6:**
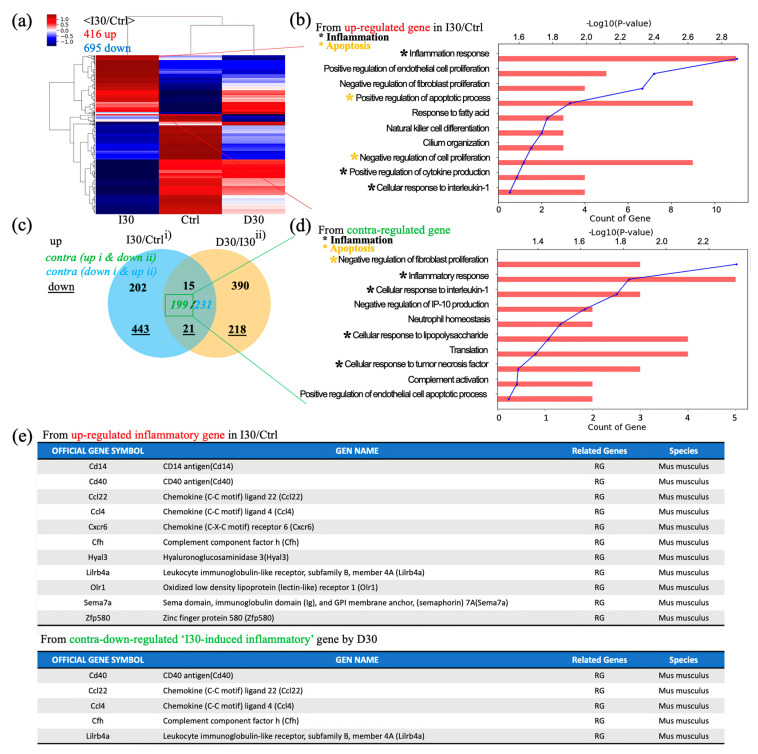
Global gene expression study using quant-sequencing to identify transcriptome features with I30, Ctrl, and D30 under osteoclast differentiation media. (**a**) Distance-based clustering analysis from global transcriptional changes in 3 groups with 1.2-fold change and over 1 log2 value from differentially expressed gene between I30 and Ctrl. The transcriptome profile with I30 differed from Ctrl and D30L. (**b**) Gene ontology (GO) analysis using up-regulated genes in I30L compared to Ctrl. Biological process analysis showed that major GO related to active inflammation and apoptosis (negative regulation of proliferation) was enriched in I30L. (**c**) Venn’s diagram of the comparison of differential gene expression based on Quant-seq data with numbers of co-up or –down and contra-regulated genes. (**d**) GO analysis using contra-regulated genes (I30L/Ctrl up-regulation & D30L/I30L down-regulation). Biological process analysis showed that major GO related to active inflammation and apoptosis (negative regulation of proliferation) were assigned in down-regulated genes by D30L, meaning a decrease of inflammation and apoptosis could occur in D30L compared to I30L. These transcriptome results support less osteoclast differentiation of precursors in D30L compared to I30L. Collectively, these results indicate that I30L stimulated osteoclast formation, while D30L suppressed the formation of osteoclasts in an in vitro study. (**e**) Inflammatory gene sets. Among 11 inflammatory up-regulated genes in I30/Ctrl, CD40, ccl22, ccl4, cfh, and lilrb4a were down-regulated by D30, supporting less osteoclastic differentiation potential of D30 than I30.

Next, we investigated overall transcriptomic differences between I30L, D30L, and the control group to investigate the underlying mechanism of why D30L had lower osteoclast formation than I30L and the control group. A heatmap profile of D30L resembled the control group more than I30L. Upregulated genes of I30L were analyzed; genes with black dots indicate inflammatory genes, while yellow dots denote apoptosis genes (Figure 6a,b). Venn’s diagram, using differentially expressed genes between groups, shows overall transcriptome expression (Figure 6c). In detail, gene ontology (GO) analysis using up- or down-regulated genes (3094) between Control + I30L and I30L + D30L presented active inflammation and apoptosis from the biological process (Figure 6d). We suspected that genes downregulated in D30L out of the upregulated genes from Control + I30L might explain why D30L showed lower osteoclast formation. Therefore, we analyzed genes upregulated in I30L and downregulated in D30L. According to GO analysis using contra-regulated genes, D30L showed lower inflammation and apoptosis genes than I30L. The exact mechanism or factor is unknown, but the effect of D30L reveals a decrease in overall inflammatory genes and apoptosis in genes compared to I30L and the control group. For supplementary, out of 10 inflammatory genes, 5 genes were downregulated in D30L. CD40 antigen, chemokine (Ccl22, Ccl4), and leukocyte immunoglobulin-like receptor (Lilrb4a) can trigger an inflammatory response. Further study is needed to identify the genes that decrease osteogenic differentiation.

## 4. Discussion

Sodium iodide paste is a suggested root-filling material in primary teeth [12]. Vitapex^®^ is one of the well-known endodontic materials for pulp treatment of primary teeth. However, iodoform, one of the components of Vitapex^®^, may cause early exfoliation of primary teeth receiving pulp therapy with Vitapex^®^. From a clinical perspective, the resorption of deciduous teeth should reflect the eruption of the successor. To compensate for the shortcomings of iodoform, sodium iodide (NaI) was selected as an iodoform replacement to develop another paste clinically similar to Vitapex^®^. NaI is a water-soluble ionic compound with the antibacterial effectiveness of iodine [12], widely used in the medical field as a diagnostic tool to evaluate thyroid function [33]. It is also administered as a supplement for total parenteral nutrition but is more commonly used in veterinary medicine [34].

The main components of Vitapex^®^ are iodoform 40.4%, calcium hydroxide 30.3%, and silicone oil 22.4% [3]. To compare Vitapex^®^ and NaI paste under the same conditions, we prepared Vitapex^®^ in the laboratory by mixing calcium hydroxide, iodide, and silicone oil (polydimethylsiloxane) with equal proportions and named it I30. NaI paste was prepared in the laboratory with calcium hydroxide, sodium iodide, and silicone oil with the same proportion as I30 and named D30. This study used three different viscosities of silicone oil: 3500 (H), 1000 (M), and 350 (L) cst. Low oil viscosity affects lubricant effectiveness and oil film formation due to the lack of stickiness of the fluid. High oil viscosity produces too sticky pastes that cannot extrude from the syringes. Therefore, we examined the physicochemical and biological properties of I30 and D30 to evaluate their potential for clinical use; the study compared osteoclast differentiation among three different silicone oils to identify pastes for clinical use with the slowest root resorption of endodontically treated primary teeth. Understanding the properties of a paste is crucial for clinical decision-making and selecting the most appropriate filling material.

D30 groups showed higher flowability results than I30 groups. The study tested flow and film thickness to ensure material consistency and workability for clinical use [35]. High flow means low paste thickness. The ability of a paste to fill accessory canals and dentinal tubules is evaluated by the flowability of the filling material [36,37,38]. D30L showed a superior flow rate than other pastes. Several factors may influence the penetration of endodontic pastes within confined areas of the root canal system. Among them, dimensional irregularities, accessibility to complexities, and flow rates of pastes play an important role in allowing paste penetration [39]. L showed superior flow values with low film thickness than H and M. Thus, D30L may penetrate irregular root canals as flowability is an essential property of endodontic pastes.

D30 and I30 showed no difference in radiopacity. The radiopacity of endodontic pastes enables them to be easily distinguishable on radiographs [40]. Our results showed that all tested pastes exceeded the minimum 3 mm Al radiopacity values determined by ISO standards. According to previous reports, iodoform showed better radiopacity than sodium iodide [12]. However, our study did not show significant results compared to sodium iodide and iodoform. Optical imaging was used to visualize the surfaces of pastes; D30 was bluish-grey, and I30 was yellow. Optical images have limitations and cannot examine the interfacial gaps and voids unless root canals are sectioned and examined. Further investigation is needed to visualize pastes in the root canal.

The solubility of D30 was significantly higher than I30. According to ISO standards, an endodontic paste should present less than 3% solubility. The solubility of I30 met the standard value when D30 exhibited higher solubility than recommended, possibly due to its hydrophilic character. Higher solubility may produce gaps between the paste and the root canal, leading to microleakage [38,41]. The high solubility of D30 might be attributable to the presence of hydrophilic particles in these pastes, which may increase surface area and sealer solubility when in contact with water/moisture [42,43].

Furthermore, higher ion release was observed in D30 compared to I30. The amount of sodium and iodine released was significantly different, with the highest values observed for D30L followed by D30M and D30H. Moreover, the highest amount of calcium ion release was observed in D30L. The cytoplasmic membrane is a vital component of bacterial cells, maintaining the integrity of the cell and regulating the transport of nutrients. However, certain external factors such as the high pH of calcium hydroxide can disrupt this delicate balance, leading to a breakdown in membrane function and ultimately cell death [44]. A high pH may lead to high antibacterial activity, where alkaline pH plays a key role in enhancing the antibacterial activity of the material, improving the healing process, and increasing the deposition of mineralized components [37,38,45]. When calcium hydroxide comes into contact with water, it breaks down into calcium and hydroxyl ions. The hydroxyl ions are believed to give calcium hydroxide its high alkalinity, which makes it effective against oral bacteria and helps with remineralization [46]. Hydroxyl ions can penetrate deep into dentin, and removing the smear layer from root canal instrumentation can help facilitate their diffusion [47]. The interference in biosynthetic processes, critical to microorganisms’ survival, can be attributed to the process of lipid peroxidation and the influence of hydroxyl ions. Therefore, the existence of hydroxyl ions can lead to bacterial enzymatic deactivation. Furthermore, compounds containing iodine are utilized in dentistry for infection control due to their potent antimicrobial activity [48]. This efficacy is attributed to the high reactivity of iodine, which enables it to precipitate proteins and oxidize essential enzymes. Moreover, the ability to release calcium and iodoform ions can enhance the bioactivity and biocompatibility of endodontic pastes. Notably, our study demonstrated a higher release of calcium and iodoform in D30 paste, which facilitates tissue regeneration by binding to both soft and hard tissues while also possessing molecular signaling properties through the use of functionalized ligands or growth factors. [49,50,51,52]. This bioactivity is evidenced by the high levels of Ca^2+^ observed in the D30 paste [45]. Furthermore, we postulate that the silicone oil L displayed the highest ion release due to its single-bond chemical structure whereas silicone oil H has a double-bond chemical structure, which may confine ions (Figure S2). Therefore, the incorporation of silicone oil L in the D30 paste can lead to high bioactivity with antibacterial effectiveness by releasing hydroxyl, iodine, and calcium ions.

D30 showed lower viscosity than I30. Endodontic paste viscosity is an important parameter determining flow characteristics [37]. The viscosity of tested pastes decreased with increased flow. Decreased complex viscosities with increased frequency indicate strong non-Newtonian behavior (pseudoplastic) [53,54]. Our study showed that the viscosity of pastes decreased with increasing frequency (shear rate), indicating pseudo-plasticity and implying that the material easily flowed when extruded through a needle [37,54]. The rheological properties were also correlated with the injection force (F) through hypodermic needles by syringeability tests. Injection force is mainly affected by the following three parameters: viscosity, injection flow rate, and needle characteristics [22,55]. The set injection speed dictates the fluid flow rate through the needle, a higher value of which increases the injection force [22]. In our study, D30L showed the lowest values for viscosity and injection force. Decreased viscosity of paste results in reduced injection force, indicating high flowability of the paste.

D30L paste penetrated more into root canals than the I30L paste when the artificial root canal was filled with pastes. It is necessary that the paste no longer flows and retains its shape immediately after application to prevent the extrusion of the material from the root canal. Mortazavi et al. reported a higher number of overfilled canals and the presence of voids with Metapex (one of the iodoform-based pastes). They attributed this finding to the thinner viscosity of the premixed paste, which may flow more easily into the narrow canals of primary molars and reach the apex or even beyond [4]. Endodontic treatment was performed in an artificial tooth to evaluate the potency of paste extrusion. D30L filled the root canal completely up to the apex without overfilling compared to other pastes. However, variations can occur from disparities in the hand strength of the practitioner. Future studies should examine the relationship between hand strength and perceived injection effort to verify our findings. D30L paste can be easily removed using an ultrasonic scaler. This property is crucial to facilitate the retreatment of primary teeth with reduced chair time for pediatric patients. D30L showed better removability due to its hydrophilicity compared to iodoform paste, enhancing root canal preparation and filling.

Since pastes may be in direct contact with periradicular tissues, they should be biocompatible [56]. Biocompatibility is the ability of a material to achieve a stable and advantageous host response during application. Most studies assess biocompatibility by cytotoxic tests [57]. Therefore, this study evaluated the cytotoxicity of two pastes that showed the best result in physicochemical properties: I30L and D30L. According to ISO standards, pastes are cytotoxic if cell viability is below 70% and both materials meet ISO standards [58]. Compared to I30L, D30L decreased the viability of macrophages by 70% when macrophages were cultured at high concentrations (100%). The reason for cell death could be attributed to the role of sodium iodide. However, higher cell cytotoxicity of D30L may indicate high antibacterial effectiveness. High antibacterial efficacy in root canals is crucial, considering root canal treatments mostly fail due to bacterial persistence in the root canal system.

Although I30L and D30L met ISO standards for cell cytotoxicity, materials may trigger inflammatory reactions or stimulate mineralization when in contact with periradicular tissues, resulting in root resorption. The relationship between root canal filling pastes and root canal-treated primary teeth has not yet been established. However, it is suggested that the initiation and regulation of root resorption are dependent upon the stellate reticulum and the dental follicle of the underlying permanent tooth, through the release of activating molecules such as cytokines and osteoclastic bone resorption transcription factors. As a result, the present study conducted an analysis of the expression of well-known osteogenic enzymes and transcription factors such as NFATc1, cathepsin K, TRAP, and c-Fos. 

Osteoclastic activity is triggered by the binding between the receptor activator of nuclear factor κB (RANK) and the receptor activator of nuclear factor κB ligand (RANKL), a key factor for osteoclast differentiation and activation [59]. It leads to the activation of the transcription factors NFATc1 and c-Fos. 

c-Fos is an essential transcription factor for the formation of osteoclasts. The nuclear factor of activated T-cells (NFATc1) drives the differentiation of the mononuclear precursors into multinucleated active osteoclasts. In 25% concentration of osteoclast differentiation, c-Fos, and NFATc1 increased significantly on days 3 and 4, respectively, compared to D30L. Moreover, tartrate-resistant acid phosphatase (TRAP)—the primary enzyme produced by osteoclasts, and cathepsin K—one of the lysosomal enzymes produced by osteoclasts [60], have been identified as major markers associated with the degradation of bone mineral and collagen matrices [61,62]. These markers were upregulated in the I30L compared to D30L in our study. This finding summarizes that the overexpression of NFATc1, c-Fos, TRAP, and cathepsin K in I30L may accelerate osteoclast differentiation, which is a mechanism of root resorption in primary teeth, whereas D30L exerts a relative inhibitory effect on these genes, indicating that D30L may delay osteoclastic formation compared to I30L. 

Studying the formation and activity of TRAP-positive is a well-established method of assessing osteoclast function and formation [63,64]. TRAP enzyme is highly expressed in osteoclasts and is typically located within the ruffled border [65,66]. In our research, we compared osteoclast formation and TRAP activity in D30L. To assess the cytoskeletal structure and function of osteoclasts, actin staining was employed. The actin cytoskeleton plays a critical role in resorption and is an essential component of osteoclasts. It is known to be dynamic, undergoing rounds of resorption with actin ring formation and movement without actin ring formation [67,68]. During resorption, osteoclasts reorganize disordered, diffused microfilaments into an elaborate belt or ring-like structure of actin rings that surrounds the ruffled plasma membrane involved in resorption [69,70]. After fixation with paraformaldehyde, fluorescent phalloidin was utilized to visualize the actin structure, and the outcomes indicated an increase in actin rings triggered by extracts of I30L, indicating the ability of cells to resorb teeth. 

T-cell activation occurs after odontoclast cells start resorbing the dentin and predentin from the inner surface under the influence of locally produced cytokines. These activated T cells then induce and activate odontoclast cells, resulting in the removal of dentin and enamel. In our study, cytokines from I30L were upregulated compared to D30L: CD 40 antigen (Cd40), chemokine ligand 22 (ccl22), chemokine ligand 4 (ccl4), complement factor H (cfh), and leukocyte immunoglobulin-like receptor subfamily B member 4a (lilrb4a). The CD40 molecule is crucial for promoting inflammatory responses by macrophages [71]. Plasma chemokines ccl22 and ccl22,4 are the most potent chemoattractants for the CD4^+^CD25^+^ T cell population [72]. The complement factor H *(*CFH*)* gene on the chromosome plays a role in innate immunity and inflammation [73]. According to previous reports, LILRB4 could be involved in a system to prevent inflammatory responses. These upregulated cytokines in I30L indicate more osteoclast differentiation, which may result in faster root resorption when the primary tooth is treated and filled with I30L compared to D30L. 

Sodium iodide-based paste reduced osteoclast differentiation compared to iodoform-based paste commercially available as Vitapex^®^. It also presented acceptable physicochemical properties (Figure S3); therefore, NaI can be used as a substitute for iodoform.

However, this study has several limitations. First, the results of in vitro experiments may not apply to in vivo conditions. The in vitro result cannot simulate clinical situations involving different biomechanical environments. Second, this study used a small sample size with limited time. This experiment has short-term follow-up considering that endodontically treated teeth retain filling material for a few years. Further investigation is required to evaluate sodium iodide as a root-filling paste in clinical settings and assess the biological response of sodium iodide. 

## 5. Conclusions

In summary, our study produced significant findings indicating that the sodium iodide root filling material with L silicone oil (D30L) is a clinically acceptable option due to its favorable physicochemical and biological properties. Compared to iodoform-based pastes, D30L demonstrated better flow, film thickness, pH, viscosity, and injection force along with improved filling ability and removability. Additionally, our study has uncovered a noteworthy result with regards to the application of D30L. Our findings indicate that the use of D30L leads to a reduction in osteoclast differentiation and mRNA expression. This result is significant as it demonstrates that the rate of root resorption in primary teeth following pulp treatment is comparable to the natural process of physiological resorption. These findings have enabled us to reject our null hypothesis and establish that D30L holds potential as a viable alternative to iodoform-based paste in the field of dentistry. Such a promising outcome provides a strong foundation for future investigations and advances in dental treatment.

## Figures and Tables

**Table 1:** Flow, film thickness, radiopacity, solubility, and viscosity at 0.5 Hz. (Mean ± SD). Within each column, the significant differences between groups are indicated by different superscript letters.

Variable	Flow (mm)	Film Thickness (µm)	Radiopacity (mm)	Solubility (%)	Viscosity at 0.5 Hz (Pa.s)
I30H	${14.4\pm \left(0.72\right)}^{a}$	${44.0\pm (6.08)}^{a}$	${8.42\pm (0.16)}^{a}$	${0.17\pm (0.02)}^{a}$	${91.1\pm \left(7.87\right)}^{c}$
I30M	${16.1\pm \left(0.08\right)}^{a}$	${33.3\pm (4.04)}^{b}$	${8.25\pm (0.36)}^{b}$	${0.178\pm (0.05)}^{b}$	${40.1\pm (9.05)}^{c}$
I30L	${17.6\pm (0.44)}^{b}$	${23.7\pm \left(3.22\right)}^{c}$	${8.49\pm (0.06)}^{c}$	${0.239\pm \left(0.03\right)}^{c}$	${22.1\pm (6.79)}^{c}$
D30H	${17.0\pm (0.27)}^{b}$	${41.0\pm \left(3.46\right)}^{c}$	${8.33\pm \left(0.13\right)}^{d}$	${11.28\pm \left(0.98\right)}^{c}$	${71.0\pm (9.99)}^{c}$
D30M	${17.5\pm (0.42)}^{c}$	${36.7\pm \left(3.22\right)}^{b}$	${8.59\pm \left(0.27\right)}^{e}$	${17.07\pm \left(2.66\right)}^{d}$	${32.6\pm \left(3.46\right)}^{c}$
D30L	${18.1\pm (0.38)}^{c}$	${30.3\pm (5.86)}^{c}$	${8.49\pm (0.06)}^{c}$	${20.01\pm (2.41)}^{c}$	$9.87{\pm (2.27)}^{c}$

Within each column, there was a significant difference between groups with different superscript letters (*p* < 0.05).

## Data Availability

Publicly available datasets were analyzed in this study.

## Supplementary Materials

The following supporting information can be downloaded at: https://www.mdpi.com/article/10.3390/pharmaceutics15041072/s1, Figure S1: Schematic figure of mixed materials; Figure S2: Chemical structure of silicone oil H and silicone oil M, L; Figure S3: Summary of overall experiments on I30L and D30L. Except for solubility, D30L exhibited better physicochemical and biological outcomes; Table S1: Manufacturer and Chemical composition of material used in present study.
