# Supplementary material for: Physicochemical, Pre-Clinical, and Biological Evaluation of Viscosity Optimized Sodium Iodide-Incorporated Paste
Source: Pharmaceutics. 2023 Mar 27;15(4):1072. doi: 10.3390/pharmaceutics15041072 (PMC10143732; doi:10.3390/pharmaceutics15041072)
Supplement: Supplementary file 1 [file pharmaceutics-15-01072-s001.zip › pharmaceutics-2266152-supplementary.pdf]

pharmaceutics

Supplemental Table S1. Manufacturer and Chemical composition of material used in present study

Materials	Composition, details	Manufacturer
I30H	Calcium Hydroxide 33.3%<br>Iodoform 33.3%<br>Silicone Oil 33.3%	Sigma–Aldrich, Burlington, MA, USA<br>Alfa Aesar, Heysham, LA 2XY, UK<br>Sylgard 184, Dow Corning Co., Midland, MI, USA
I30M	Calcium Hydroxide 33.3%<br>Iodoform 33.3%<br>Silicone Oil 33.3%	Sigma–Aldrich, Burlington, MA, USA<br>Alfa Aesar, Heysham, LA 2XY, UK<br>Shin-Etsu Silicone KF-96, Shin-Etsu Chemical Co., Tokyo, Japan
I30L	Calcium Hydroxide 33.3%<br>Iodoform 33.3%<br>Silicone Oil 33.3%	Sigma–Aldrich, Burlington, MA, USA<br>Alfa Aesar, Heysham, LA 2XY, UK<br>Shin-Etsu Silicone KF-96, Shin-Etsu Chemical Co., Tokyo, Japan
D30H	Calcium Hydroxide 33.3%<br>Sodium Iodide 33.3%<br>Silicone Oil 33.3%	Sigma–Aldrich, Burlington, MA, USA<br>Sigma–Aldrich, Burlington, MA, USA<br>Sylgard 184, Dow Corning Co., Midland, MI, USA
D30M	Calcium Hydroxide 33.3%<br>Sodium Iodide 33.3%<br>Silicone Oil 33.3%	Sigma–Aldrich, Burlington, MA, USA<br>Sigma–Aldrich, Burlington, MA, USA<br>Shin-Etsu Silicone KF-96, Shin-Etsu Chemical Co., Tokyo, Japan
D30L	Calcium Hydroxide 33.3%<br>Sodium Iodide 33.3%<br>Silicone Oil 33.3%	Sigma–Aldrich, Burlington, MA, USA<br>Sigma–Aldrich, Burlington, MA, USA<br>Shin-Etsu Silicone KF-96, Shin-Etsu Chemical Co., Tokyo, Japan

Proportional ratios

	Iodoform	Sodium iodide	Calcium hydroxide	Silicone oil
I30	10		10	10
D30		10	10	10

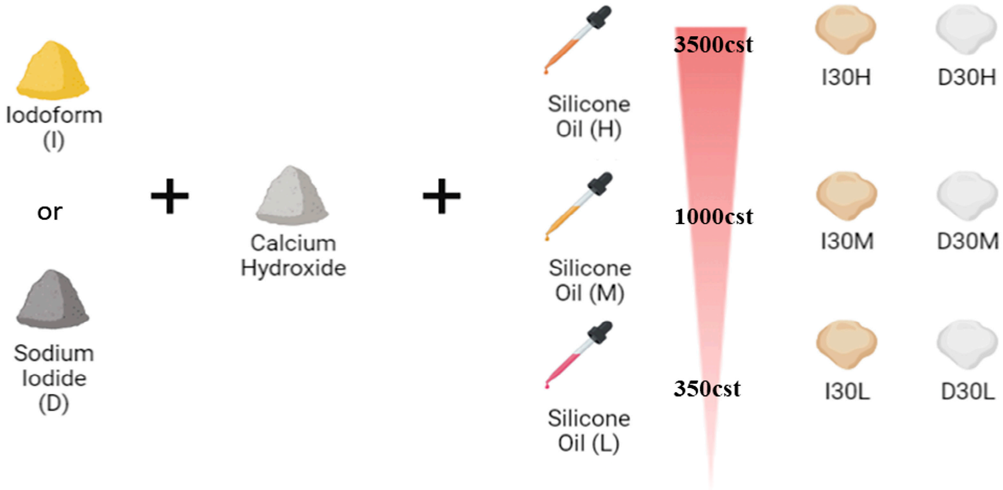

Supplemental Figure S1. Schematic figure of mixed materials.

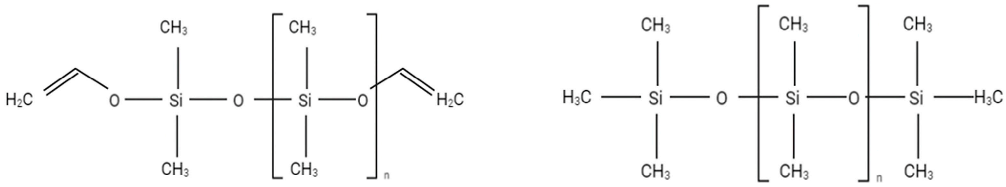

Sylgard 184 silicone elastomer (Polydimethylsiloxane) **KF-96 (dimethylpolysiloxane)**

Supplemental Figure S2. Chemical structure of silicone oil H and silicone oil M, L.

	I30L	D30L
Flow	-	↑
Film Thickness	-	↓
Radiopacity	-	-
Solubility	↓	-
pH	-	↑
Ion release	-	↑
Viscosity	-	↓
Injection Force	-	↓
Filling Ability	-	↑
Filling Removability	-	↑
Cell Viability	↑	-
Osteoclast Differentiation	-	↓
mRNA expression	-	↓

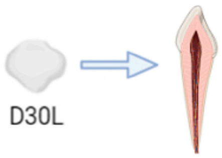

Supplemental Figure S3. Summary of overall experiments on I30L and D30L. Except for solubility, D30L exhibited better physicochemical and biological outcomes.
